# A new species of *Aplectana* Railliet & Henry, 1916 (Nematoda: Cosmocercidae) in the Brazilian Amazon and the taxonomic status of *Aplectana longa*

**DOI:** 10.1590/S1984-29612023074

**Published:** 2023-12-04

**Authors:** Ana Nunes Santos, Ewerton dos Santos Borges, Yuri Willkens, Jeannie Nascimento dos Santos, Carlos Eduardo Costa-Campos, Francisco Tiago de Vasconcelos Melo

**Affiliations:** 1 Laboratório de Biologia Celular e Helmintologia “Profa. Dra. Reinalda Marisa Lanfredi”, Instituto de Ciências Biológicas, Universidade Federal do Pará – UFPA, Belém, PA, Brasil; 2 Laboratório de Herpetologia, Departamento de Ciências Biológicas e da Saúde, Universidade Federal do Amapá – UNIFAP, Macapá, AP, Brasil

**Keywords:** Nematodes, microscopy, first record, Aplectana, Nematódeos, microscopia, primeiro registro, Aplectana

## Abstract

Nematodes of the genus *Aplectana* Railliet & Henry, 1916 are common parasites of the digestive tract of amphibians and reptiles in the Neotropical region. During a parasite survey on *Boana boans* (Linnaeus, 1758), we found specimens of nematodes with *Aplectana* characteristics. We observed a set of characteristics that differs the species of our study from its congeners, and the present study describes a new species of *Aplectana* parasite of *B. boans* using light microscopy and scanning electron microscopy. *Aplectana pella* n. sp. has lateral alae and somatic papillae in males and females; males have equal short spicules, and the gubernaculum is absent. The arrangement of pairs of caudal papillae also differs from other species (2 precloacal,1 adcloacal, and 5 postcloacal+1 unpaired). In females, the vulva is simple, with non-prominent lips, and equatorial. This is the first record of the genus *Aplectana* parasitizing *B. boans* and the 58^th^ species described for this genus. Additionally, we added the precloacal papillae pattern of *A. delirae,* and based on morphological and morphometric characteristics, we propose the reallocation of *Aplectana longa* to the genus *Oxyascaris*.

## Resumo

Nematódeos do gênero *Aplectana* Railliet & Henry, 1.916, são parasitos comuns do trato digestório de anfíbios e répteis na região Neotropical. Durante um levantamento de parasitos em *Boana boans* (Linnaeus, 1.758), foram encontrados espécimes de nematódeos com características de *Aplectana*. Observou-se um conjunto de características que diferencia a espécie deste estudo de outros congêneres, e o presente estudo descreve uma nova espécie de *Aplectana* parasito de *B. boans*, usando-se microscopia de luz e microscopia eletrônica de varredura. *Aplectana pella* n. sp. possui alas laterais e papilas somáticas em machos e fêmeas; os machos têm espículos curtos e iguais e gubernáculo ausente. O arranjo de pares de papilas caudais o diferencia de outras espécies (2 pré-cloacais, 1 adcloacal e 5 pós-cloacais +1 ímpar não pareada). Nas fêmeas, a vulva é simples, sem lábios proeminentes, e situada na região equatorial do corpo. Este é o primeiro registro do gênero *Aplectana* parasitando *B. boans* e a 58ª espécie descrita para esse gênero. Além disso, foi adicionado o padrão de papilas pré-cloacais de *A. delirae* e, com base em características morfológicas e morfométricas, foi proposta a realocação de *Aplectana longa* para o gênero *Oxyascaris*.

## Introduction

Nematodes of the family Cosmocercidae Railliet, 1916 are frequently found parasitizing the digestive tract of amphibians and reptiles worldwide. Among the members of Cosmocercidae, the most common genera found in hosts from the Neotropics are species of *Aplectana* Railliet & Henry, 1916 and *Cosmocerca* Diesing, 1861, with *Aplectana* being the most abundant among neotropical amphibians and richest in species (Bursey et al., 2001; Campião et al., 2014; Bursey et al., 2011; González et al., 2021).

Currently, there are 57 described species in the genus *Aplectana,* of which 28 species are distributed throughout the Neotropics (Ramallo et al., 2007; Sou et al., 2018; Alcantara et al., 2021). Species of the genus *Aplectana* that have already been reported infecting *Boana* spp. are *Aplectana* sp. in *Boana pardalis* (Spix, 1824) in the Goiás state, Brazil, and in *Boana* sp. (location not reported); *Aplectana travassosi* (Gomes & Motta, 1967) in *Boana raniceps* (Cope, 1862) in Ceará state, Brazil and Paraná state, Brazil; and *Aplectana* sp. in *Boana albopunctata* (Spix, 1824) in Brasília, Brazil (Travassos, 1925; Holmes et al., 2008; Graça et al., 2017; Machado et al., 2022).

The genus *Boana* Gray, 1825, comprises anuran species distributed throughout Central and South America, from Nicaragua to Argentina, and are also present in Trinidad and Tobago (Frost, 2023). To date, no species of the genus *Aplectana* have been found in *Boana boans* (Linnaeus, 1758). Therefore, this study describe a new species of the genus *Aplectana* parasitizing *B. boans* in the Brazilian Amazon. Additionally, after analyzing *Aplectana* spp. from the Neotropics for morphological comparisons, we noted some disparities in the identification of *Aplectana longa* Alcantara & Silva, 2021. Thus, we proposed a new combination and relocation to the genus *Oxyascaris* Travassos, 1920.

## Materials and Methods

Amphibian samples were collected within the “Beija-Flor Brilho de Fogo” Extractive Reserve, located in the southern part of the municipality of Pedra Branca do Amapari, state Amapá, Brazil (0°47 '30.6' N, 51°58 '42.1' W). The region comprises dense forests with large trees, with the Amapari River as the main watercourse, and the climate is equatorial (Pedroso-Santos et al., 2019). The collections were performed through an active search (visual and auditory) during June and September 2021, totalizing 16 specimens of *B. boans* (SISBIO: No 48102-2).

The frogs were euthanized with lidocaine (2%) and necropsied to remove all internal organs, which were later transferred to Petri dishes containing NaCl solution (0.9%). The nematodes were killed in heated 70% alcohol and preserved in tubes containing 70% alcohol. For morphological and morphometric analyses, the helminth specimens were hydrated in distilled water, clarified with Amman’s Lactophenol, mounted on temporary slides, and analyzed under an Olympus BX41 microscope equipped with a drawing tube. The classification of helminths was performed using catalogs and identification keys, based mainly on Anderson et al. (2009), Gibbons (2010), and other relevant scientific articles.

Four specimens (two males and two females) were selected for analysis by scanning electron microscopy (SEM) and subjected to post-fixation in 1% Osmium tetroxide (OsO_4_) and then washed in distilled water. Subsequently, they were dehydrated in an increasing series of ethanol (30% to 100%), dried in a CO_2_ Critical Point, then mounted on aluminum metal supports, and sputtered with gold-palladium. The specimens were analyzed under a Tescan Vega3 microscope in the Laboratory of Structural and Functional Biology (LABEF – ICB), Federal University of Pará (UFPA), Brazil.

For additional morphological comparisons, we examined holotypes and paratypes of *Aplectana delirae* deposited in the Helminthological Collection of the Oswaldo Cruz Institute, Brazil (CHIOC), under deposit numbers CHIOC 30591 a-l.

The measurements are presented as the holotype/allotype values, followed by the minimum and maximum in parentheses (reported in micrometers unless otherwise indicated). The prevalence, mean abundance, and mean intensity were calculated according to Bush et al. (1997) and Reiczigel et al. (2019). The specimens were deposited in the Collection of Other Invertebrates of the “Museu Paraense Emílio Goeldi” (MPEG), municipality of Belém, state of Pará, Brazil.

## Results

### Taxonomic summary

#### Superfamily Cosmocercoidea Railliet, 1926

#### Family Cosmocercidae Railliet, 1925

#### Subfamily Cosmocercinae Railliet, 1916

#### Genus *Aplectana* Railliet & Henry, 1916

#### *Aplectana pella* n. sp. Santos, Borges & Melo

*Type-host: Boana boans* (Linnaeus, 1758) (Amphibia: Hylidae)

*Type-locality*: “Beija-Flor Brilho de Fogo” Extractive Reserve, municipality of Pedra Branca do Amapari, Amapá, Brazil (0º47'30.6”N, 51º58'42.1' 'W)

*Deposited specimens*: MPEG 00281 (Holotype); MPEG 00282 (allotype); MPEG 00283 (paratypes females); MPEG 00284 (paratypes males)

*Site of infection*: Small intestine

*Prevalence*: 25% (4/16)

*Mean intensity*: 6.5

*Mean abundance*: 1.63 analyzed host

*Registration of ZooBank*: urn:lsid:zoobank.org:pub:A9BB3BA8-CF6F-4F98-A4C8-D748D0FD04B5

*Etimology*: The specific epithet is after the species of hummingbird *Topaza pella* (Linnaeus, 1758), known as the “Beija-Flor Brilho de Fogo” which gives rise to the name of the “Beija-Flor Brilho de Fogo” Extractive Reserve.

#### Description (Figures 1-2)

**Figure 1:**
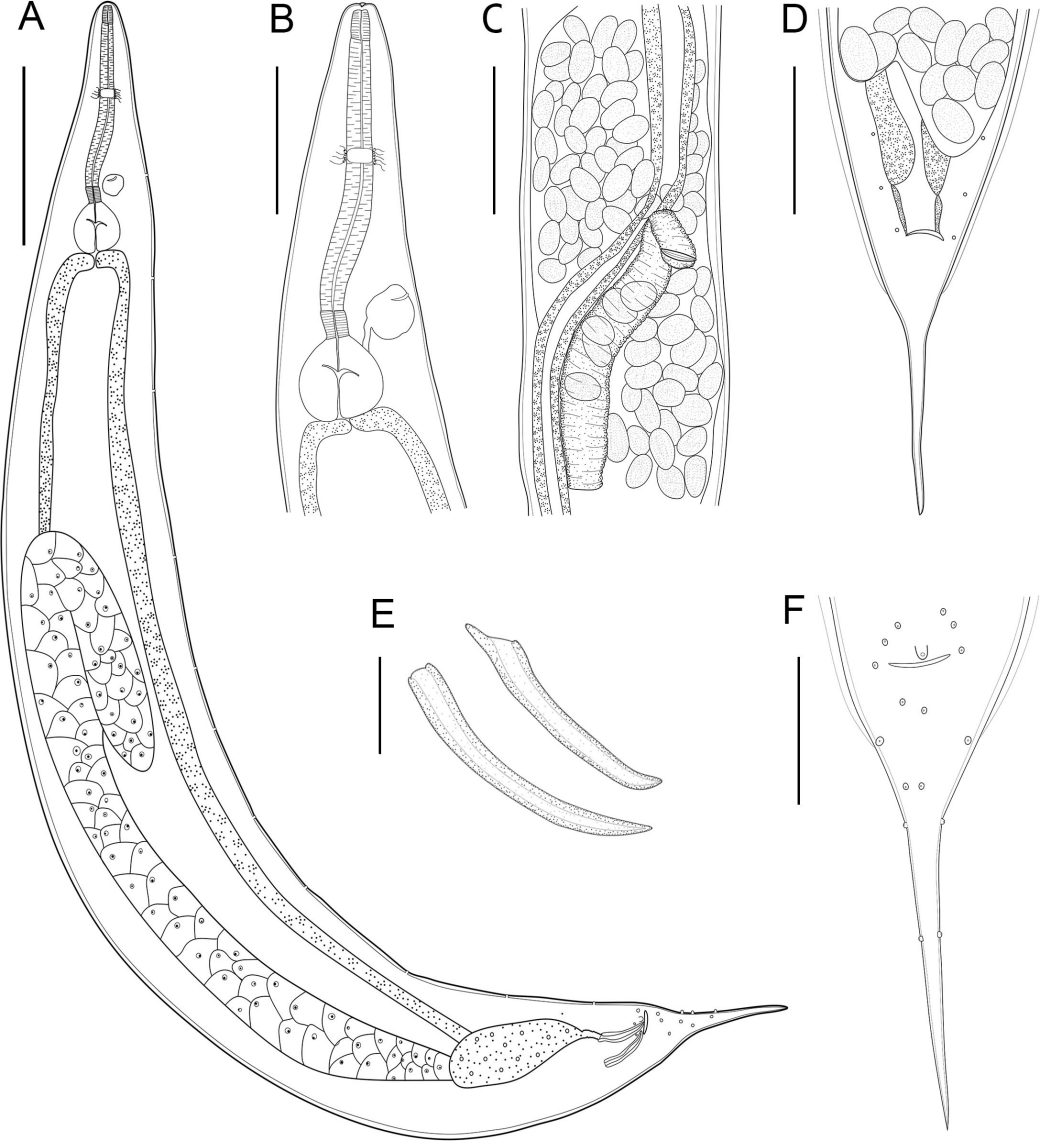
*Aplectana pella* n. sp. holotype and allotype line drawings. **(A)** Male, general view; **(B)** Female anterior end, lateral view; **(C)** Female reproductive system; **(D)** Female posterior end, ventral view; **(E)** Spicules; **(F)** Male posterior end, ventral view. *Scale-bars*: A: 350 μm; B, C, D: 200 μm; E: 100 μm; F: 25 μm.

**Figure 2:**
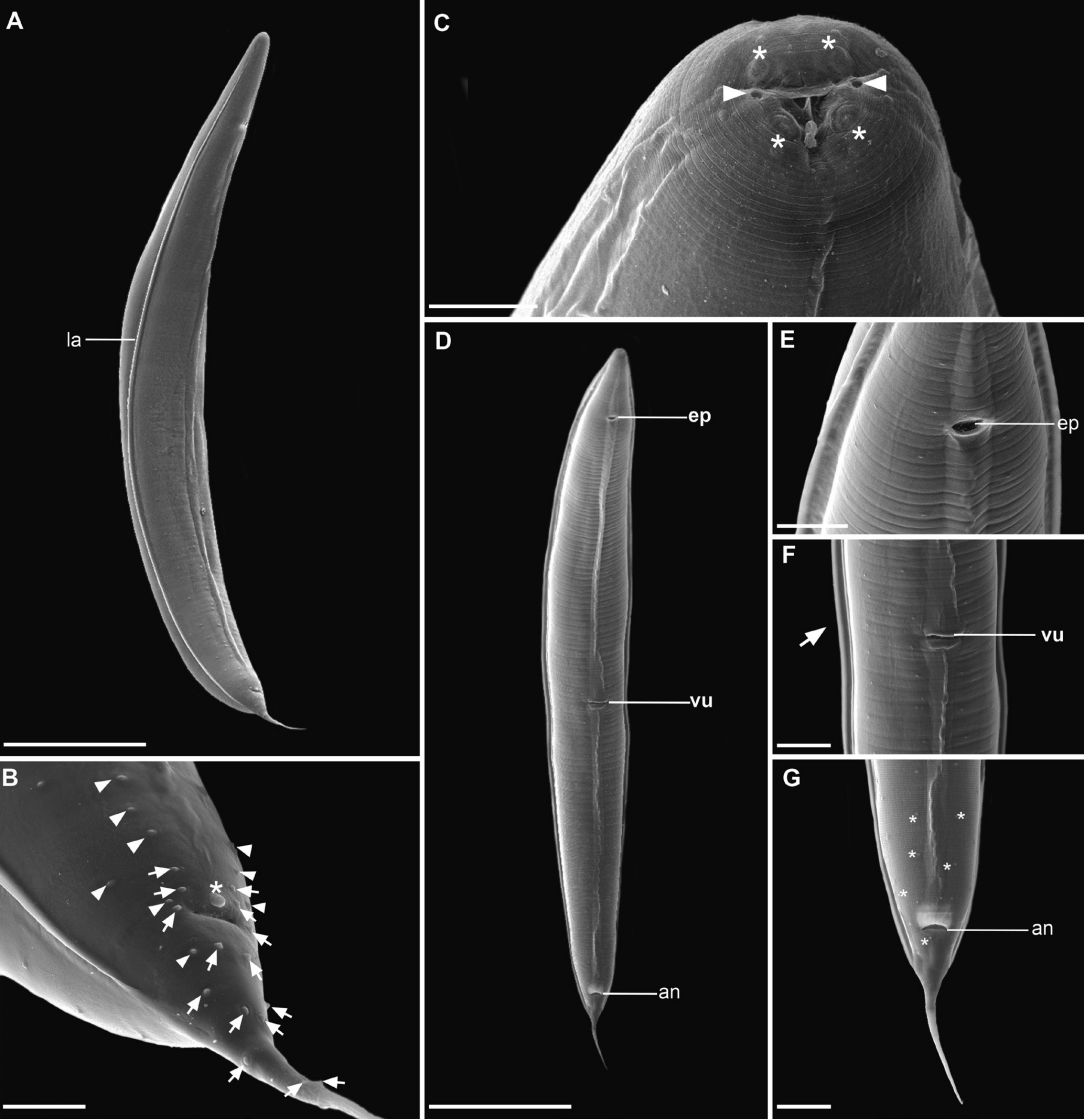
Scanning electron microscopy of *Aplectana pella* n. sp. **(A)** Male general view, ventro-lateral; Lateral alae (la); **(B)** Male posterior end, lateral view; cloacal papillae (arrow), somatic papillae (arrowhead), unpaired papillae (*); **(C)** Female anterior end, cephalic papillae (*) amphids (arrowhead); **(D)** Female, ventral view, excretory pore (ep), vulva (vu), anus (an); **(E)** Female, detail of excretory pore (ep); **(F)** Female, detail of vulva (vu), lateral alae (arrow); **(G)** Female posterior end; anus (an), somatic papillae (*). *Scale-bars*: A, D: 500 μm; B, E: 50 μm; C: 20 μm; F, G: 100 μm.

*General*. Small-sized nematodes. Cuticle with fine transverse striations. Sexual dimorphism evident, males smaller than females. Lateral alae present in both sexes, beginning anterior to nerve ring and ending near tail tip (Figure 22F). Oral aperture simple, triangular, surrounded by 3 small lips, dorsal lip with one pair of papillae, subventral lips with one papilla and one amphid each (Figure 2C). Esophagus divided into anterior short pharynx, cylindrical corpus, slightly narrower isthmus, posterior bulb with valves (Figure 1B). Excretory pore (Figures 1B, 22E) slightly anterior to isthmus. Somatic papillae small, with small and non-prominent sensilla in both sexes distributed along whole-body length, very evident in tail of females (Figures 11D, 22G). Tail conical with a filament in both sexes.

**Males (*based on 1 holotype and 6 paratypes*):** Body 2.7 (2.3−2.9) mm long (Figure 2A). Width at esophagus-intestinal junction 226 (205−251). Esophagus total length 486 (416−502), pharynx 34 (27−42) × 32 (26−32), corpus 325 (269−330) long, isthmus 34 (32−45), bulb 93 (77−98) × 122 (101−122). Nerve ring from anterior end 221 (173−226.5), excretory pore from anterior end 341 (312−344). Posterior end of body distinctly curved ventrally (Figure 22B). Spicules small, curved, similar in shape and length (Figure 1E) 111 (104–111) long. Gubernaculum absent. Caudal papillae with more prominent sensilla and distributed as: 2 pairs ventral precloacal near anterior clocal lip; 1 pair adcloacal; 5 pairs of postcloacal papillae (first and third pairs ventral, second sublateral, and fourth and fifth pairs lateral); 1 single unpaired papilla situated on anterior cloacal lip (Figures 1 F, 2B). Tail 320 (256–344) long.

**Female (*based on 1 allotype and 9 paratypes*):** Body 3.5 (2.3–4.3) mm long (Figure 2D). Body width at vulva level 358 (289–436). Width at esophagus-intestinal junction 242 (178–306). Esophagus 592 (529–594) long (Figure 1B), pharynx 48 (32–50) × 32 (26–37). Corpus 377 (336–413) long, isthmus 40 (32–50) long. Bulb 112 (96–117) × 133 (114–144). Nerve ring 202 (178–245) from anterior end (Figure 1B). Excretory pore situated 408 (349–453) from anterior end (Figure 2D). Vulva equatorial, with simple and non-prominent vulvar lips, vulvar aperture as a transversal slit 1.6 (0.6–2) mm from anterior end (Figures 1 C, 22F). Vagina muscular, directed anteriorly and flexed posteriorly. Ovaries two, located anterior to vulva. Uterus with numerous elliptical eggs (Figure 1C), surrounded by a sclerotized shell, at different stages of development 54 (54–67) × 33 (33–43) (based on 10 eggs). Tail 332 (321–394) long (Figures 1 D, 2G)

### Remarks

*Aplectana* species are grouped mainly by the presence or absence of an unpaired papilla on the anterior cloacal lip and the presence or absence of the gubernaculum. In addition, other important characteristics that distinguish the species of the genus are the number and arrangement of caudal papillae, and the length of spicules (Gomez et al., 2017).

*Aplectana pella* n. sp. belongs to the group of species in which the gubernaculum is absent, listed below: *A. vercammeni* Le Van Hoa, 1962, *A. akhrami* (Islam, Farooq & Khanum, 1979), *A. dubrajpuri* Sou & Nandi, 2015, *A. artigasi* Puga & Torres, 1997, *A. chilensis* Lent & Freitas, 1948, *A. crossodactyli* (Vicente & Santos, 1970) Baker, 1980, *A. crucifer* Travassos, 1925, *A. delirae* (Fabio, 1971) Baker, 1980, *A. meridionalis* Lent & Freitas, 1948, *A. papillifera* Araujo, 1977 and *A. tarija* Ramallo, Bursey & Goldberg, 2007. At the same time, *A. artigasi*, *A. chilensis*, *A*. *crossodactyli*, *A. crucifer*, *A. delirae*, *A. meridionalis* and *A. papillifera* recorded in the Neotropics; while *A. vercammeni* has been reported in the Ethiopian region, and *A. akhrami* and *A. dubrajpuri* are from Oriental Region (Fabio, 1971; Baker, 1980; Ramallo et al., 2007; Sou & Nandi, 2015).

Besides *A. vercammeni*, *A. akhrami* and *A. dubrajpuri* have not been recorded in hosts in the Neotropical region, the new species shares the characteristic of having an unpaired papilla on the anterior cloacal lip only with *A. dubrajpuri* (Sou & Nandi, 2015). However, *Aplectana pella* n. sp. and *A. dubrajpuri* are easily differentiated by the distribution pattern of caudal papillae pairs (pre:ad:postcloacal + unpaired), 2:1:5+1 in *A. pella vs*. 3:1:14+1 in *A. dubrajpuri*; by total body length (2.3–2.9 mm for males and 2.3 to 4.3 mm for females *vs.* 1.06–1.15 mm for males and 1.37–1.51 mm for females, respectively); by spicule length (104–111 *vs.* 140–160, respectively) and vulvar position (equatorial *vs.* post equatorial, respectively) (Sou & Nandi, 2015).

Among the Neotropical species in which the gubernaculum is absent, *Aplectana pella* n. sp. resembles *A. artigasi* because it has an unpaired papilla on the anterior cloacal lip. However, it differs in the following characteristics: the new species is smaller compared to *A. artigasi* (2.3−2.9 mm for males and 2.3−4.3 mm for females *vs.* 3−4 mm *vs.* 3.8−6 mm, respectively); the pattern of caudal papillae pairs (2:1:5+1 *vs.* 14:1:10-14+1, respectively), the total number of pairs of papillae in the new species is smaller (8 in *Aplectana pella* n. sp. *vs*. 25 – 29 in *A. artigasi*); the length of the spicules is shorter in *Aplectana pella* n. sp. (104–111 *vs.* 322–364, respectively) and have a shorter distance from the vulva to the anterior end (0.6–2 mm *vs.* 2.3–3.3 mm, respectively). We also observed that the eggs in the new species are smaller than those in *A. artigasi* (54–67 × 33–43 *vs.* 69−124 × 41−82.8, respectively) (Puga & Torres, 1997).

*Aplectana chilensis* is easily differentiated from *Aplectana pella* n. sp. because the latter has an unpaired papilla on the anterior cloaca lip (absent in *A. chilensis*). In addition, the species have different number and arrangement of caudal papillae pairs (2:1:5+1 in the new species *vs*. 12:0:6 in *A. chilensis*), and the spicules are smaller in the new species (104–111 in *Aplectana pella* n. sp. *vs.* 260–410 in *A. chilensis*), as well as the tail length of females (321–394 in *Aplectana pella* n. sp. *vs.* 640–670 in *A. chilensis*) (Lent & Freitas, 1948).

The new species resembles *A. crossodactyli* because both have an unpaired papilla on the anterior lip of the cloaca. However, these species differ in the number and arrangement of caudal papillae pairs (2:1:5+1 in the new species *vs.* 20:0:5+1 in *A. crossodactyli*), the spicules are smaller in the new species (104–111 in *Aplectana pella* n. sp. *vs.* 130–140 in *A. crossodactyli*), males are smaller in total body length (2.3−2.9 mm *vs.* 2.8–3.7 mm in *A. crossodactyli*), as well as *Aplectana pella* n. sp. have a greater distance from the excretory pore to the anterior end in males than that *A. crossodactyli* (312−344 *vs.* 290, respectively) and the total length of the esophagus is shorter in females of the new species than that *A. crossodactyli* (529–594 *vs.* 610, respectively). Additionally, females have a shorter distance from the vulva to the anterior end (0.6–2 mm *vs.* 2.1–2.7 mm, respectively) (Vicente et al., 1990).

*Aplectana pella* n. sp. shares with *A. crucifer* the absence of the gubernaculum. However, the new species has an unpaired papilla on the anterior cloacal lip, which is absent in *A. crucifer*. Additionally, they differ by the number and arrangement of the cloacal papillae pairs (2:1:5 +1 in new species *vs*. 6:1:4 in *A. crucifer*); the length of the spicules of *Aplectana pella* n. sp. is three times smaller than that of *A. crucifer* (104–111 *vs.* 330, respectively), and the tail of females of the new species is small than that *A. crucifer* (321–394 *vs.* 560, respectively) (Travassos, 1931).

*Aplectana delirae* is a species that has been described as parasitizing anurans from the Neotropics and does not have a gubernaculum. The description by Fabio (1971) does not include details about the number and pattern of precloacal papillae but rather characterizes them only as “variable”. However, when analyzing the type series of *A*. *delirae*, we observed that males have the following pattern of caudal papillae pairs: 3 precloacal, 1 adcloacal and 4 postcloacal, and 1 unpaired papilla in the upper cloacal lip (3:1:4+1). Thus, the new species can be distinguished from *A. delirae* because it has a different pattern of caudal papillae pairs: 2:1:5+1. In addition, the males of the new species are smaller in total body length (2.3−2.9 mm in *Aplectana pella* n. sp. *vs* 3.97–4.04 mm in *A. delirae*), distance from the vulva to anterior is shorter (0.6−2 mm in *Aplectana pella* n. sp. *vs.* 2.13 mm in *A. delirae*) and the length of the spicules are smaller (104–111 in the new species *vs.* 150–170 in *A. delirae*) (Fabio, 1971).

The new species resembles *A. meridionalis* in the absence of the gubernaculum and by having an unpaired papilla on the anterior lip of the cloaca. However, they differ in the length and width of the eggs (54–67 × 33–43 in *Aplectana pella* n. sp. *vs.* 74–93 × 37–56 in *A. meridionalis*), distance from nerve ring to the anterior end in males (176– 226 in the new species *vs.* 150–190 in *A. meridionalis*) and because the spicule length is shorter in the new species (104–111 *vs.* 130–136). Additionally, they differ by the pattern of cloacal papillae pairs (2:1:5+1 in *Aplectana pella* n. sp. *vs*. 7:2:5+1 in *A. meridionalis*) (Lent & Freitas, 1948).

*Aplectana papillifera* resembles the new species by having an unpaired papilla on the anterior lip of the cloaca, and the gubernaculum is absent. However, they can be differentiated by the number and arrangement of the caudal papillae pairs. *Aplectana pella* n. sp. has a pattern of 2:1:5+1, while in *A. papillifera,* the pattern is 10:1:8+1. In addition, the new species has shorter spicules (104–111 in the new species *vs.* 400 in *A. papillifera*), a smaller body length in both males and females (2.3−2.9 mm in males and 2.3−4.3 mm in females *vs.* 4.4–5.2 mm in males and 5.9–8.5 mm in females, respectively) and a shorter distance from the vulva to the anterior end (0.6–2 mm *vs.* 3.5–5 mm, respectively) (Araujo, 1976/77).

Finally, *Aplectana pella* n. sp. resembles *A. tarija* in the absence of gubernaculum and by having an unpaired papilla on the anterior lip of the cloaca. However, they can be easily distinguished by the number and arrangement of the cloacal papillae pairs (2:1:5+1 *vs.* 1:0:4+1, respectively), the spicule is shorter in the new species (104–111 *vs.* 110–120, respectively), females with shorter body lengths (2.3–4.3 mm *vs.* 4.7–5.8 mm, respectively) and shorter distance from the vulva to the anterior end (0.6–2 mm *vs.* 2.7–3.3 mm, respectively) (Ramallo et al., 2007).

## Discussion

### Species distinction and taxonomic status of *Oxyascaris longum* n. comb.

In the present study, the nematodes described have lateral alae, somatic papillae, male caudal papillae without rosettes or plectanes, females with two ovaries anterior to the vulva, and numerous thin-shelled eggs in the uterus. According to Baker (1980) and Gibbons (2010), these are characteristics commonly exhibited by species of the genus *Aplectana*.

*Aplectana pella* n. sp. can be included in the group of species that do not have a gubernaculum and in which males have an unpaired papilla on the anterior cloacal lip. Currently, *Aplectana* includes 57 nematode species parasitic of amphibians and reptiles that are distributed worldwide, of which 16 have been recorded in Brazil (Bursey et al., 2018; Alcantara et al., 2021). In the present study, we did not include *Aplectana longa* Alcantara and Silva, 2021 in species comparisons because the morphological and morphometric characteristics described by the authors demonstrate that the species may have been mistakenly allocated to the genus.

*Aplectana longa* is a species described as parasitizing *Gastrotheca microdiscus* (Andersson, 1910) in São Paulo, Brazil. Among the main characteristics proposed for the species, the authors highlight the absence of lateral alae and the total length of both males and females, at that time, being one of the largest species of the genus. In addition, they add the absence of lateral alae to the diagnosis of the genus *Aplectana* (Alcantara et al., 2021). However, the lateral alae has been widely used as a diagnostic feature of *Aplectana* [see Baker (1980), Anderson et al. (2009), Gibbons (2010)], and it is noteworthy that this would be the only species of the genus known thus far that does not have lateral alae. In addition, according to Baker & Vaucher (1985), mature females of *Aplectana* spp. do not exceed 10 mm, it is noteworthy that in *A. longa*, females measure 10.7–20.8 mm and they presented numerous eggs and larval development in the uterus, similar to *Oxyascaris* spp.

The genera of the family Cosmocercidae are differentiated mainly through the type of ornamentation associated with papillae in the caudal end of males (Anderson et al., 2009). The genera *Aplectana*, *Oxyascaris*, *Oxysomatium* and *Raillietnema* share the same type of caudal papillae (simple and unadorned). In *Oxysomatium*, *Raillietnema,* and *Oxyascaris* females have a characteristic reproductive system (amphidelphic) and are easily differentiated from *Aplectana*. In addition, Baker & Vaucher (1985) proposed that large females and numerous eggs or free larvae in the uterus are characteristics of *Oxyascaris*. Thus, carefully observing the specimens described by Alcantara et al. (2021), we propose that *Aplectana longa* should be relocated to the genus *Oxyascaris*.

Notably, Freitas (1958) pointed out that in the genus *Oxyascaris*, the bulb is reduced in width. Thus, when analyzing the five valid species of the genus *Oxyascaris* (*O. caatingae* Félix-Nascimento, 2020; *O. caudacutus* (Freitas, 1958); *O. oxyascaris* Travassos, 1920; *O. similis* (Travassos, 1920); and *O. mcdiarmidi*Bursey & Goldberg, 2007, we observed, empirically (see Supplementary Table S1) and through the line drawings presented, that the bulb is smaller in *Oxyascaris* spp. than in *Aplectana* spp. [see Freitas (1958), Bursey & Goldberg (2007), Felix-Nascimento et al. (2020)]; similarly to *A. longa* esophageal bulb.

Additionally, we calculated the bulb width/body length ratio of females and males of *Oxyascaris* spp. and Neotropical species of *Aplectana*. When calculating the proportion for the species of *Aplectana*, we observed that the bulb ratio varied from 1.74% – 6% in males and 0.80%–6% in females. On the other hand, the bulb width/body length ratio for *Oxyascaris* varied from 0.95%–2.15% in males and 0.48%–0.85% in females. Thus, we concluded that in the species of *Aplectana,* the bulb is larger than that of *Oxyascaris* spp. Detailed comparisons among bulb width/total length are presented in Supplementary Table S1.

According to the illustration and morphometry presented by Alcantara et al. (2021), the bulb of *A. longa* is less dilated than that of other *Aplectana* species. After calculating the bulb width/total length proportion, we found that this structure corresponds to 1.6% of the body length in males and 0.65% in females, similar to *Oxyascaris* spp. In addition, the body length of the females and the absence of the lateral alae are additional evidence that cast doubt on the allocation of *A. longa* to the genus.

We conclude that based on the morphological characteristics of the esophagus, the absence of lateral alae, the body length of female specimens, and the presence of eggs and larvae in the uterus of females of *A. longa*, the species should be reallocated to the genus *Oxyascaris* and named *Oxyascaris longum* n. comb.

To validate *Oxyascaris longum* n. comb., we compared the species with its congeners. We will use the following set of characteristics to differentiate the species: number and distribution of caudal papillae, spicule length, presence/absence of gubernaculum, presence/absence of lateral alae, presence/absence of unpaired papilla in the cloacal anterior lip and position of the vulva in relation to the anterior end of the female’s body. According to Freitas (1958), Baker & Vaucher (1985), and Felix-Nascimento et al. (2020), these are the main characteristics that differentiate *Oxyascaris* spp.

*Oxyascaris longum* n. comb. differs from all *Oxyascaris* species in terms of the number and distribution of caudal papillae 9:0:6 (precloacal: adcloacal: postcloacal), a pattern that is not observed in the other species. Another noteworthy feature is the spicule length (232–258), as it is longer than *O. caatingae* (95–109), *O. caudacutus* (122–134), *O. oxyascaris* (150–206), and *O. similis* (150–206) and shorter than in *O. mcdiarmidi* (612–689).

Additionally, the lateral alae are absent in *O. longum* and are present in *O. oxyascaris*, O. *caudacutus, O. similis,* and *O. mcdiarmidi*. *Oxyascaris longum* n. comb. is the only species of the genus with a vulva in the equatorial position. In the other species of the genus, the vulva is in the pre-equatorial region of the body (*O. caatingae*, *O. caudacutus*, *O. similis,* and *O. oxyascaris*) and post-equatorial (*O. mcdiarmidi*). Thus, *O. longum* n. comb. becomes the 6th known species of *Oxyascaris*.

## Final Remarks

The nematodes analyzed in our study have characteristics corresponding to the genus *Aplectana* and represent a new species, which was described using light microscopy and SEM. In the present study, we reallocated *A. longa* to the *Oxyascaris longum* n. comb. proposing 6^th^ species of the genus *Oxyascaris.*

Furthermore, this is the first record of the genus *Aplectana* for the hylid *B. boans*. Thus, we describe the 58^th^ species of *Aplectana*, the 28th species for the Neotropics, and the 17^th^ species for Brazil, adding new data on the diversity of helminth parasites of frogs in the Brazilian Amazon.

Conflict of interest

## Supplementary Material

Supplementary material accompanies this paper.

Table S1 Measurements of total body length, width of esophageal bulb and bulb width/total length ratio of *Oxyascaris* spp. and Neotropical species of *Aplectana*.

This material is available as part of the online article from https://doi.org/10.1590/S1984-29612023074
